# “Teamwork in hospitals”: a quasi-experimental study protocol applying a human factors approach

**DOI:** 10.1186/s12912-017-0229-z

**Published:** 2017-06-29

**Authors:** Randi Ballangrud, Sissel Eikeland Husebø, Karina Aase, Oddveig Reiersdal Aaberg, Anne Vifladt, Geir Vegard Berg, Marie Louise Hall-Lord

**Affiliations:** 1 Department of Health Science Gjøvik, Faculty of Medicine and Health Sciences, Norwegian University of Science and Technology, Gjøvik, P.O. Box 191, 2802 Gjøvik, Norway; 2 Department of Health Studies, Faculty of Social Sciences, University of Stavanger, P.O. Box 8600 Forus, , 4036 Stavanger, Norway; 3 Department of Surgery, Stavanger University Hospital, Gerd Ragna Bloch Thorsens street 8, 4011 Stavanger, Norway; 4 Innlandet Hospital Trust Division Lillehammer, Lillehammer, Norway; 5 Department of Health Sciences, Faculty of Health, Science and Technology, Karlstad University, Karlstad, Sweden

**Keywords:** Inter-professional team training, Intervention, Hospital, Human factors, Patient safety, Teamwork

## Abstract

**Background:**

Effective teamwork and sufficient communication are critical components essential to patient safety in today’s specialized and complex healthcare services. Team training is important for an improved efficiency in inter-professional teamwork within hospitals, however the scientific rigor of studies must be strengthen and more research is required to compare studies across samples, settings and countries. The aims of the study are to translate and validate teamwork questionnaires and investigate healthcare personnel’s perception of teamwork in hospitals (Part 1). Further to explore the impact of an inter-professional teamwork intervention in a surgical ward on structure, process and outcome (Part 2).

**Methods:**

To address the aims, a descriptive, and explorative design (Part 1), and a quasi-experimental interventional design will be applied (Part 2). The study will be carried out in five different hospitals (A-E) in three hospital trusts in Norway. Frontline healthcare personnel in Hospitals A and B, from both acute and non-acute departments, will be invited to respond to three Norwegian translated teamwork questionnaires (Part 1). An inter-professional teamwork intervention in line with the TeamSTEPPS recommend Model of Change will be implemented in a surgical ward at Hospital C. All physicians, registered nurses and assistant nurses in the intervention ward and two control wards (Hospitals D and E) will be invited to to survey their perception of teamwork, team decision making, safety culture and attitude towards teamwork before intervention and after six and 12 months. Adult patients admitted to the intervention surgical unit will be invited to survey their perception of quality of care during their hospital stay before intervention and after six and 12 month. Moreover, anonymous patient registry data from local registers and data from patients’ medical records will be collected (Part 2).

**Discussion:**

This study will help to understand the impact of an inter-professional teamwork intervention in a surgical ward and contribute to promote healthcare personnel’s team competences with an opportunity to achieve changes in work processes and patient safety.

**Trial registration:**

Trial registration number (TRN) is ISRCTN13997367. The study was registered retrospectively with registration date 30.05.2017.

## Background

On the basis of international studies, the World Health Organization (WHO) estimates that between 3 and 16% of all patients treated in hospitals are affected by adverse events [1]. Poor teamwork is an independent cause of many of the system failures that lead to patient harm [2–4], which are considered as preventable [5, 6]. Despite improvements in the past 15 years, patient safety remains an important public health concern [7]. Effective teamwork and good communication are critical components essential to patient safety in today’s specialized and complex healthcare services [3, 8, 9]. A focus on teamwork competencies in complex healthcare systems such as leadership and coordination are identified by the WHO as a priority for research on patient safety in the Western world [10]. Teamwork is described in terms of behaviour, cognitions and attitudes that make interdependent performance possible [11], which is defined as: “The interaction or relationship of two or more health professionals who work interdependently to provide care for patients” ([12], p. 3). Teamwork is one of six core competencies seen as necessary for health professionals to master in order to meet the current and future demands for the quality of care [13].

Hospital units are representing frontline microsystems in hospitals where patients and healthcare professionals meet, and where care quality, patient safety and clinical outcomes are being produced [14]. These microsystems have the greatest opportunity to develop and improve work processes [15]. An extensive review of factors associated with team performance has identified shared mental models, mutual respect and trust, and closed-loop communication as the underpinning conditions required for effective teams [16]. However, various challenges exist within the frontline healthcare environment. For example, there are different perceptions and expectations of the roles and collaboration in teams among healthcare professionals [17, 18]. Moreover, studies have also demonstrated a variation in their perception of patient safety culture [19, 20], while another challenge to effective teamwork is the hierarchical structure among healthcare personnel [21, 22]. The association between adverse events and insufficient teamwork have been known for some time, but it is only in recent years that an evidence-based understanding of the problem has been developed, as well as an efficient programme to promote patient safety. Furthermore, it has been demonstrated that team training can improve efficiency in inter-professional teamwork within hospitals [23–26]. Team training is recommended for those who are expected to work together in teams [27], which requires that health-care personnel across professions should train together. Although there is an increasing awareness regarding teamwork competencies, healthcare has implemented team training in healthcare education and clinical practice to a small extent [28–30]. Team Strategies and Tools to Enhance Performance and Patient Safety (TeamSTEPPS®) [31] is an evidence-based teamwork system based on research on teamwork, team training and cultural change [16, 32, 33]. The teamwork system is released from the US Agency for Healthcare Research and Quality (AHRQ) and the Department of Defense as a national team training programme. TeamSTEPPS provides tools, strategies and measurements to promote team practice in all aspects of healthcare [34], and with the use of an implementation strategy based on Kotter’s model of organizational change [35].

### Theoretical framework

The Systems Engineering Initiative for Patient Safety 2.0 model (SEIPS 2.0) [36, 37], a framework developed from a human factors perspective for patient safety, will be used as a theoretical framework for this research project on teamwork in hospital. Human factors is a discipline concerned with the understanding of the interaction between humans and other elements of a system. According to Health and Safety Executive [38], human factors refer to: environmental, organizational and job factors, and human and individual characteristics which influence behaviour at work in a way that can affect health and safety. The general structure of the SEIPS 2.0 model includes the work system, which produces work processes, which in turn shapes the outcomes [36]. Frontline healthcare personnel are doing their work in an inter-professional patient care team in the centre of a complex work system in which structural factors such as practical tasks/procedures, equipment and technology, physical environment and organization affect their teamwork, and thus the results related to safety and quality in patient care [39]. A human factor-based healthcare system redesign with the SEIPS model is found in a systematic review [40] as a useful approach for improving patient safety and quality of care.

### Rationale for the research project

Since the majority of patient medical treatment and care takes place in inter-professional teams, the quality of the teamwork is a key feature of patient safety. Despite previous research shows that interventions focusing on inter-professional team training promote the quality of professional practice, there are still little knowledge about its impact on hospitals wards. Most research on teamwork and team training has been conducted in operating rooms, intensive care and emergency rooms. Furthermore, most research on teamwork has been conducted in the United States, but less in European and Scandinavian. This study “Teamwork in hospitals” will be conducted in hospital wards among frontline healthcare personnel in Norway. The patient care teams within these units consist of inter-professionals team members with a common goal and responsibility for a defined groups of patients.

### Aim

The aims of this study are to translate and validate teamwork questionnaires and investigate healthcare personnel’s perception of teamwork in hospitals (Part 1), and to explore the impact of an inter-professional teamwork intervention in a surgical ward on structure, process and outcome (Part 2).

## Methods/design

### Design

In order to address the aims of the study, a descriptive, explorative and a quasi-experimental interventional design will be applied. The study comprises six sub-studies and an overview of the specifics aims, designs and methods is shown in Table 1.

**Table 1:** Overview of the six sub-studies, aims, designs and methods

	Part 1		Part 2			
	Study 1	Study 2	Study 3	Study 4	Study 5	Study 6
Aims	To translate questionnaires into Norwegian and to analyse the questionnaire’s psychometric properties.	To investigate healthcare personnel’s perception of teamwork and team decision making, and attitude towards teamwork.	To explore the impact of an inter-professional teamwork intervention in a surgical ward with regard to team decision making, patient safety culture and teamwork.	To describe inter-professional team members’ and leaders’ perception of teamwork and the impact of implementation of a teamwork intervention in a surgical ward.	To explore patients’ perception of quality of care in relation to an inter-professional teamwork intervention in a surgical ward.	To explore the impact of an inter-professional teamwork intervention in a surgical ward with regard to patient outcome and adverse events.
Design	Cross-sectional	Cross-sectional	Quasi-experimental – controlled before-and- after.	Qualitative descriptive	Quasi-experimental – uncontrolled before-and-after.	Quasi-experimental – time-series
Intervention			Six hours of inter-professional team training and implementation of tools and strategies in a surgical ward.			
Sample/Setting	Healthcare personnel, approximately 600, working in acute and non-acute units in Hospital A and Hospital B.	Healthcare personnel, approximately 600, working in acute and non-acute units in Hospital A and Hospital B.	All healthcare personnel, approximately 120, working in three surgical wards in three hospitals, including one intervention ward (Hospital C) and two control wards (Hospital D and Hospital E).	Leaders, physicians, registered nurses and assistant nurses, approximately 20, working in the intervention surgical ward in Hospital C.	Patients admitted to the intervention surgical ward (Hospital C) who meet the inclusion criteria: 18 years or older, understand Norwegian, are in a mental and physical health condition which makes it ethically justifiable to participate. Power analysis is conducted.	Patient register data and data from patients’ medical records (Hospital C). A power analysis with regard to number of patients’ medical records is conducted.
Data collection	Translation and back-translation of the three questionnaires: Teamwork Perceptions Questionnaire (T-TPQ), Teamwork Attitude Questionnaire (T-TAQ), Collaboration and Satisfaction About Care Decisions (CSACD). A survey with a paper version of the translated questionnaires.	A survey with a paper version of the translated questionnaires T-TAQ, T-TPQ, CSACD.	An electronic survey with T-TAQ, T-TPQ, CSACD and HSOPSC questionnaires will be administered at three occasions (before the intervention, and after six and 12 months). Ward statistics will be collected.	Focus group interviews will be conducted on three occasions (before the intervention, and after six and 12 months).	A survey with paper version of the questionnaire Quality from Patient’s Perspective will be administered on three occasions (before the intervention and after six and 12 months).	Anonymous patient data from local registers and from medical records (by use of Global Trigger Tool) will be released before and during the intervention period.
Data analysis	IBM SPSS 23 with descriptive statistics, explorative factor analysis and IBM SPSS AMOS 22 with confirmatory factor analysis. Intraclass Correlation (ICC) and Cronbach’s alpha	IBM SPSS 23 with descriptive statistics, Chi-square test, Mann-Whitney U-test, Kruskal-Wallis test, t-test, ANOVA and Multiple regression analysis.	IBM SPSS 23 with descriptive statistics, Chi-Square test, Mann-Whitney U-test, Kruskal-Wallis test, Paired t-test, Wilcoxon Signed rank test, ANOVA and Multiple regression analysis.	A Manifest inductive content analysis.	IBM SPSS 23 with descriptive statistics, Chi-Square test, Mann-Whitney U-tests, Kruskal-Wallis test, ANOVA and Multiple regression analysis.	IBM SPSS 23 with descriptive statistics, Time series analysis. Chi-Square test, Mann-Whitney U-tests, Kruskal-Wallis tests, ANOVA and Multiple regression analysis.

### Research setting

The study will be carried out in five different hospitals (Hospital A, B, C, D, E) in one hospital trust in Norway, and will represent both acute and non-acute departments. Hospital A is represented with internal medicine, gynaecology, obstetrics, surgery, emergency room, intensive care, operating rooms and anaesthesia, and Hospital B with internal medicine (Part 1). Hospital C is represented with a surgical intervention ward and Hospital D and Hospital E with one surgical control ward each (Part 2).

### Sample and data collection

#### Healthcare professionals

The target population is frontline healthcare personnel.

In part 1, all physicians (110), registered nurses (405), assistant nurses (59), midwives (24), physiotherapists (19) and occupational therapists (7) will be invited as respondents. Three Norwegian translated questionnaires measuring teamwork will be tested for psychometric properties, and healthcare personnel’s perception of teamwork, team decision-making and attitude towards teamwork will be investigated. A paper-based version of the Teamwork Perceptions Questionnaire (T-TPQ) [41], Teamwork Attitude Questionnaire (T-TAQ) [42], Collaboration and Satisfaction About Care Decisions (CSACD) [43] and questions on sociodemographic and educational background will be administered to the frontline healthcare personnel.

In part 2, all physicians, registered nurses and assistant nurses in an intervention ward and two control wards will be invited as respondents to explore the impact of an inter-professional teamwork intervention in a surgical ward with regard to team decision-making, patient safety culture and teamwork. An electronic survey with the Norwegian translated questionnaires T-TPQ [41], CSACD [43], T-TAQ [42], Hospital Survey on Patient Safety Culture (HSOPSC) [44] and data on sociodemographic and educational background will be administered to the frontline healthcare personnel before intervention, and after six and 12 months. Focus group interviews with physicians (*n* = 5), registered nurses (*n* = 5), assistant nurses (*n* = 5) and leaders (*n* = 4) in the surgical intervention ward will be conducted before intervention, and after six and 12 months, to explore their perception of teamwork and the impact of the inter-professional teamwork intervention within the ward.

#### Patients

In part 2, patients admitted to the intervention surgical ward who meet the inclusion criteria: being 18 years or older, understand Norwegian, as well as being in a mental and physical health condition that makes it ethically justifiable to participate, will be invited to explore their perception of quality of care during their hospital stay. A paper version of the questionnaire Quality from Patient’s Perspective (QPP) [45] will be administered to the patients before the intervention, and after six and 12 months. Patient size calculation was estimated by power analysis. To detect a mean difference of 0.4 (the primary endpoint) of the item “participation in medical treatment” a sample size of 65 (baseline), 80 (after 6 months) and 80 (after 12 months) (alpha <.05, power 0.80, standard deviation of 0.9) would be needed to find a significant difference between groups.

#### Patient register data and data from patients’ medical records

In part 2, anonymous patient registry data from local registers and data from patients’ medical records will be collected before and during the intervention period. Sample size with regard to patients’ medical records was estimated by power analysis. The proportion of patients is estimated on the variable readmission within 30 days. Based on previous measurements in the hospital the number of patients readmitted within 30 days in the surgical ward was assumed to be 20% (4 of 20 reviewed patient records). To detect a difference of 10% in rates between baseline and during the implementation (alpha <.05, power 0.90) a total 532 patient records will be reviewed. Patient registry data refers to incidence of falls, decubitus ulcers, infections, postoperative complications and hospital stays and re-admission within 30 days will be followed before and during the intervention.

#### Ward statistics

Data from ward statistics will be collected from the intervention ward and the control wards. Examples of variables are “number of physicians, registered nurses, assistant nurses, beds, frequency of over-occupancy”.

#### Intervention

The inter-professional teamwork intervention in a surgical ward at Hospital C (Part 2) is planned according to the TeamSTEPPS-recommended “Model of Change” [31], and is organized into three phases: Phase 1) Setting the stage and deciding what to do - Assessment; Phase 2) Making it happen - Training and implementation; and Phase 3) Making it stick - Monitoring, integrating, and providing coaching for the initiatives to be sustained over time [31]. One day of team training consisting of 4 h classroom training (lectures, videos, role-plays and discussions) and 2 h of high-fidelity simulation for all healthcare personnel in the surgical intervention ward will be conducted. To ensure the quality of the educational programme, the classroom training and simulation training will be piloted. The team training will be carried out by four trainers (nurses and physicians) from the intervention ward in collaboration with members of the research group. A strategy for further implementation of the teamwork system into clinical practice will be conducted by an inter-professional change team with members from the surgical intervention ward. Moreover, all the trainers have completed the AHRQ TeamSTEPPS 2.0 Master Training Course, a two-day in-person course with a train-the-trainer approach.

### Data analysis plan

Data analysis will be performed using IBM SPSS Statistics 23 and IBM SPSS AMOS 22. Descriptive statistics will be used for displaying the frequencies, percentages, means or medians, and standard deviation and a 95% confidence interval will be reported where relevant. An explorative factor analysis (EFA) and confirmatory factor analysis (CFA) will be conducted to test the translated questionnaires for psychometric properties. Possible associations between variables will be evaluated by hypothesis tests and/or generalized linear models and generalized estimating equations to compare the results over time, and an inductive qualitative content analysis [46] will also be conducted to analyse qualitative data.

### Study status

Data collection related to Part 1 of the study was completed in December 2015, though no results have been published. With regard to Part 2, the intervention of one-day team training for healthcare personnel in the surgical intervention ward was conducted in May 2016, while the implementation of the TeamSTEPPS framework is ongoing. Surveys of healthcare personnel and patients have been carried out in April 2016 and November 2016, and will be conducted in June 2017. A retrospective data collection related to patient register data from local registers and review of medical patients records with Global Trigger Tool will start in May 2017 and is planned to be finished in December 2017.

## Discussion

The process of developing and evaluating complex interventions has a number of stages, although they may not follow a linear structure; however, the best practice is to develop interventions in a systematic way [47]. A first step is to identify existing evidence of similar interventions, in addition to which methods have been used to evaluate them [47]. This study consists of two parts with six sub-studies, each with a protocol based on a review of the literature. In the overall study protocol, the evidence is based on recent systematic reviews on teamwork and team training in hospitals. A theoretical framework and a theoretical understanding are needed to prevent weak links and to identify strengths. The SEIPS 2.0 model [36] provides a valuable lens to the theoretical understanding, which assists our research on inter-professional teamwork in a number of ways: 1) to reframe how to observe and monitor inter-professional teamwork, and interpret aspects of teamwork performance; 2) to explore more deeply what contextual factors influence the healthcare team’s performance; 3) to measure the effect of inter-professional teamwork on patient safety and patient outcomes, and 4) to help move the discussion beyond the teamwork training events (i.e. using simulation), and to provide evidence-based recommendations on the content, duration and frequency of teamwork training programmes associated with the clinical evidence [26].

It is important to understand the context in which the intervention is taking place. One challenge may be that there is still no consensus about a single definition or model of teamwork that can accommodate every feature of teamwork within a specific healthcare specialty [3]. Moreover, healthcare personnel may not be aware of the core competencies of teamwork [48, 49]. Therefore, a feasibility testing of the translated teamwork questionnaire conducted in a hospital setting is important. Moreover, the TeamSTEPPS programme [31] has to be translated and adapted into a Norwegian hospital setting.

The intervention in this study will be investigated using a quasi-experimental interventional design, which is useful where there are practical or ethical barriers to conducting a randomized experiment [50], although under an quasi-experimental design researchers have less control over confounding factors [50]. The intervention is taking place in a hospital ward, which is a small microsystem with a limited sample size that hampers the use of randomization. Quasi-experimental designs may include controlled and uncontrolled before-and-after studies and time-series design [50]. Such designs are often found in studies of improvement [51], and will also be used in this project. The decision making with regard to outcome measures is based on identifying existing evidence about similar interventions. Qualitative data from interviews will be used in process evaluations, and will contribute to exploring the way in which the intervention is implemented. It is important to understand how and why changes have taken place [51], and why an intervention has unexpected consequences or fails, or how a successful intervention works [47].

The intervention has been planned according to the TeamSTEPPS-recommended change model, and uses an implementation strategy based on Kotter’s model of organizational change. With the understanding that complex interventions may work best if they are tailored to local contexts [52], a project group comprising members of the research group and leaders from clinical practice is in collaboration responsible for the development. Moreover, a change team at the surgical intervention ward is responsible for the implementation in clinical practice.

The major strength of this study is its status as an intervention conducted in a real-world hospital setting with an opportunity to achieve changes in work processes and patient safety. However, there are methodological limitations in terms of isolation on the effect of the ongoing intervention.

## Abbreviations

2008 MRC: The new Medical Research Council guidance
AHRQ: Agency for Healthcare Research and Quality
CSACD: Collaboration and Satisfaction About Care Decisions
HSOPSC: Hospital Survey on Patient Safety Culture
QPP: Quality from Patient’s Perspective
SEIPS 2.0: The Systems Engineering Initiative for Patient Safety 2.0 model
TeamSTEPPS: Team Strategies and Tools to Enhance Performance and Patient Safety
T-TAQ: Teamwork Attitude Questionnaire
T-TPQ: TeamSTEPPS Teamwork Perception Questionnaire
WHO: The World Health Organization
